# Four butyrolactones and diverse bioactive secondary metabolites from terrestrial *Aspergillus flavipes *MM2: isolation and structure determination

**DOI:** 10.1186/2191-2858-2-9

**Published:** 2012-03-01

**Authors:** Mohamed MS Nagia, Mohammad Magdy El-Metwally, Mohamed Shaaban, Soheir M El-Zalabani, Atef G Hanna

**Affiliations:** 1 Division of Pharmaceutical Industries, Chemistry of Natural Compounds Department, National Research Centre, El-Behoos st. 33, Dokki, Cairo 12622, Egypt; 2 Microbial Activity Unit, Microbiology Department, Soil & Water and Environment Research Institute, ARC, Giza, Egypt; 3 Institute of Organic and Biomolecular Chemistry, University of Göttingen, Tammannstrasse 2, D-37077 Göttingen, Germany; 4 Pharmacognosy Department, Faculty of Pharmacy, Cairo University, Cairo, Egypt

**Keywords:** *Aspergillus flavipes *MM2, butyrolactones, biological studies

## Abstract

The chemical constituents and biological activities of the terrestrial *Aspergillus flavipes *MM2 isolated from Egyptian rice hulls are reported. Seven bioactive compounds were obtained, of which one sterol: ergosterol (**1**), four butyrolactones: butyrolactone I (**2**), aspulvinone H (**3**), butyrolactone-V (**6**) and 4,4'-diydroxypulvinone (**7**), along with 6-methylsalicylic acid (**4**) and the cyclopentenone analogue; terrien (**5**). Structures of the isolated compounds were deduced by intensive studies of their 1D & 2D NMR, MS data and comparison with related structures. The strain extract and the isolated compounds (**1**-**7**) were biologically studied against number of microbial strains, and brine shrimp for cytotoxicity. In this article, the taxonomical characterization of *A. flavipes *MM2 along with its upscale fermentation, isolation and structural assignment of the obtained bioactive metabolites, and evaluate their antimicrobial and cytotoxic activities were described.

## 1. Background

**Figure 1:**
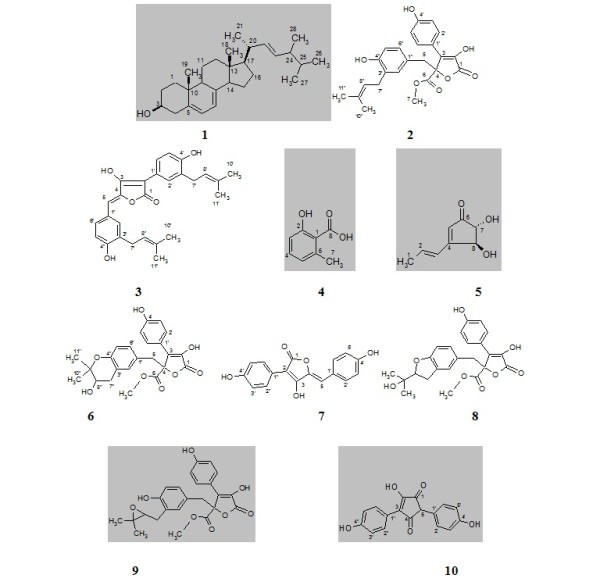
**Structural formula of the investigated compounds (1-10)**.

In recent years, numerous metabolites possessing uncommon structures and potent bioactivity have been isolated from strains of bacteria and fungi collected from diverse environments, such as soils, animals, plants and sediments [1,2]. It was noted until Alexander Fleming discovered penicillin G from *Penicillium notatum *almost 83 years ago (1928) that fungal microorganisms suddenly became a hunting ground for novel drug leads [3,4]. Therefore, many pharmaceutical companies and research groups were motivated to start sampling and screening large collections of fungal strains for antibiotics [3,5,6]. Antimycotics [7,8], antivirals [9], anticancers [10] and pharmacologically active agents [11]. The Aspergilli represents a large diverse genus, containing ca. 180 filamentous fungal species, of substantial pharmaceutical and commercial values [12]. In the research program to explore promising bioactive secondary metabolites from fungi, the terrestrial fungi, *Aspergillus flavipes *sp. isolate MM2 obtained from rice hulls, was investigated. The strain extract revealed the presence of promising antimicrobial activity against some pathogenic test organisms. Chemical screening (TLC investigation) of the strain extract showed numerous characteristic bands. Therefore, the strain was applied to large-scale fermentation by using Czapeck-Dox medium [13]. Working up of the strain cells produced ergosterol (**1**), while the filtrate extract afforded six diverse metabolic compounds: butyrolactone-I (**2**), aspulvinone H (**3**), 6-methylsalicylic acid (**4**), terrien (**5**), butyrolactone-V (**6**) and 4,4'-diydroxypulvinone (**7**). The chemical structures of the isolated compounds (**1**-**7**) were identified with the help of NMR (1D & 2D) and mass spectrometry (ESI, EI, HRESIMS) (Figure 1). The antimicrobial activity was tested against some microorganisms and cytotoxicity was examined by using brine shrimp.

## 2. Results and discussion

### 2.1. Taxonomical characterization of the fungal strain

The grown colonies of the fungal strain on Czapek-Dox medium showed bright whit-faint yellow colonies on the agar plate with a brown staining background [13]. The colonies are growing rather slowly, showing whitish from conidial masses, with brownish conidiophores shining through, reverse yellow-brown to red brown conidial heads spas, loosely columnar, conidiophores smooth-walled, pale yellow to light brown 2.4-3.2 μm in diameter. According to its morphological and microscopic characteristics and comparison with the taxonomical keys of Raper and Fennel [14], the strain was assigned as *A. flavipes *MM2.

### 2.2. Fermentation, working up and isolation

Based on the pre-screening study, the fungal strain *A. flavipes *MM2, cultivated on Czapeks-Dox for 10 days at 28°C, was shown to exhibit biological and chemical interest results. Therefore, the fungal strain was scaled up as 10 L culture using the same cultivating conditions applied for screening studies. After harvesting, both supernatant and mycelial cake phases were individually worked up. Purification of the mycelial extract using silica gel column, followed by washing the afforded major fraction by methanol and purification with Sephadex LH-20, yielded ergosterol (**1**). An application of the culture filtrate extract of *A. flavipes *MM2 to silica gel column chromatography, followed by diverse chromatographic techniques, resulted in the isolation of six compounds: butyrolactone-I (**2**), aspulvinone H (**3**), methylsalicylic acid (**4**), terrine (**5**), butyrolactone-V (**6**) and 4,4'-diydroxypulvinone (**7**).

### 2.3. Chemical characterization

#### 2.3.1. Ergosterol (1)

Ergosterol (**1**) was obtained as colourless solid, showing UV activity during TLC, which turned violet on spraying with anisaldehyde/sulphuric acid and changed latter to blue. Structure of **1 **was confirmed by different spectroscopic means (EI MS, ^1^H, ^13^C/APT NMR), chromatographic and comparison with literature [15,16]. Ergosterol plays an important role as inhibitor of lipid per-oxidation and showed strong DPPH radical scavenging activity as well [17,18], along with its cytotoxicity against HL-60 cells [19], MCF-7 cell line [20].

#### 2.3.2. Butyrolactone-I (2)

The molecular weight of **2 **was established as 424 Dalton by ESI MS, having the corresponding molecular formula C_24_H_24_O_7 _and 13 unsaturation bonds. ^1^H/H,H COSY NMR spectra of **2 **showed two o-doublets (*J *~ 8.8 Hz) each of 2H at *δ *7.57 and 6.86, being for 1,4-disubstituted aromatic residue, along with three signals at *δ *6.50, 6.48 and 6.40 representing 1,3,4-trisubstituted aromatic ring. A triplet signal of 1H was at *δ *5.05, representing an olefinic methine linked to a doublet methylene signal appeared at *δ *3.06. A 3H methoxy group (3.76); doublet of an AB methylene group (δ 3.42) attached to *sp*^2 ^system; and further two singlet methyls were visible at δ 1.65 and 1.56, representing a prenyl system.

**Figure 2:**
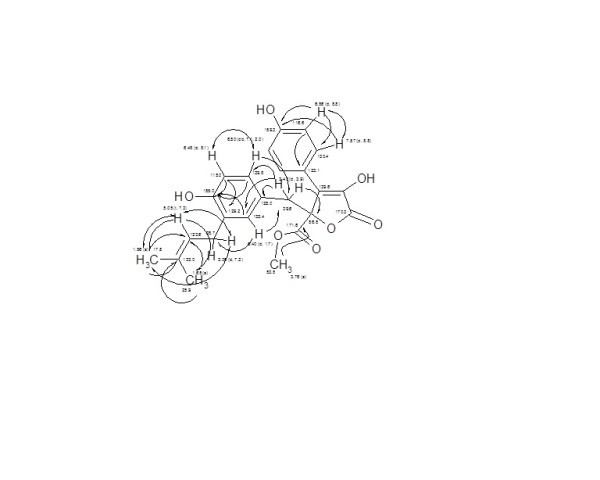
**H,H COSY (↔) and HMBC (→) correlations of butyrolactone-I (2)**.

According to the ^13^C NMR/HMQC spectra of compound **2**, 22 carbon signals representing 24 carbons were displayed, including 2 carbonyls (*δ *171.6 and 170.3), 2 *sp*^2 ^oxygenated carbons (*δ *159.3, 155.0) of phenolic systems, along with 5 quaternary carbons (*δ *139.6-123.1). Two 2CH *sp*^2 ^methine signals (130.4 and 116.6) for 1,4-disubstituted benzene ring beside to four *sp*^2 ^methines (*δ *132.4-115.0). In the aliphatic region, signals for quaternary oxygenated methine (δ 86.8), methoxy (53.8), two methylenes (*δ *39.6, 28.7) and two methyls (*δ *25.9, 17.8) were assigned. Finally, structure of **2 **was further deduced on the basis of HMBC experimental data, and comparison with literature as Butyrolactone-I [21,22]. Butyrolactone-I (**2**) was reported as a lipid lowering agent of Lovastatin × [19,23,24], showing antiproliferative activity against colon, pancreatic carcinoma, human lung cancer and prostatic cancer [25-30] (Figure 2).

#### 2.3.3. Aspulvinone H (3)

Based on the ESI MS, the molecular weight of **3 **was deduced as 432 Dalton, and the corresponding molecular formula as C_27_H_28_O_5_, containing 14 unsaturation bonds, as closely related to butyrolactone-I (**2**). ^1^H NMR spectrum of **3 **showed six confused doublets each of 1H between δ 7.81 and 6.73, being of two unsymmetrical tri-substituted aromatic residues, and singlet methine at δ 6.22. A multiplet of 2H (δ 5.36), 4H of two attached *sp*^2^-bounded methylenes (δ 3.40-3.00) and multiplet signal (δ 1.75, 12H) of four *sp*^2^-linked methyls, assigning two prenyl systems. Based on the revealed NMR data and molecular formula, and search in AntiBase [2], structure of **3 **was fixed as aspulvinone H [31].

#### 2.3.4. 6-Methylsalicylic acid (4)

According to the ESI mass spectra, the molecular weight of **4 **was deduced as 152 Dalton. The ^1^H NMR spectrum displayed three 1H resonating signals in the aromatic region (7.07, 6.63, 6.59), being of 1,2,3-trisubstiuted aromatic ring (*J *~ 7.5-8.2 Hz) together with a singlet 3H of an aromatic bounded methyl (*δ *2.56). Based on the ^13^C NMR spectrum, compound **4 **displayed eight carbon signals, including one quaternary (*δ *176). Two further deep field quaternaries were visible (*δ *162.7 and 142.7) for a-*peri*-hydroxy and methyl-*sp*^2 ^attached carbons, respectively. Three *sp*^2 ^methines (*δ *132.2, 122.9, 115.1), one quaternary (*δ *119.5) and a methyl signal (*δ *23.3). In accordance, 2-Hydroxy-6-methyl-benzoic acid (**4**) was recognized [32,33] as antifungal substance [34], in addition to its analgesic [35], herbicidal [36] and antiacne activities [37].

#### 2.3.5. Terrein (5)

The molecular weight of **5 **was deduced as 154 Dalton (C_8_H_10_O_3_), bearing four double bond equivalents. The ^1^H NMR/H,H COSY spectrum of **5 **showed two signals at δ 6.82 and 6.42 (*J *~ 15 Hz), representing a *trans*-olefinic double bond, attached to a doublet methyl (δ 1.97), constructing a terminal propene system. A further singlet (δ 5.99) being of an olefinic methine and two doublets each of 1H (δ 4.67, 4.07, *J *~ 2.8 Hz) corresponding to adjacent oxy-methines. According to ^13^C NMR/HMQC spectra, eight carbon signals were displayed, including an acetophenone carbonyl (*δ *205.6) and a deep field *sp*^2 ^quaternary carbon (δ 170.8); three *sp*^2 ^methines (δ 141.8, 126.4 and 125.9), two *sp*^3 ^oxy-methines (δ 82.4, 78.1) and one *sp*^2^-bounded methyl group (19.5).

**Figure 3:**
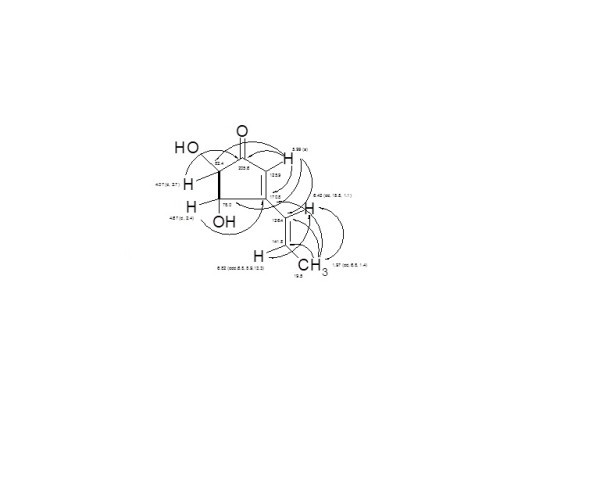
**H, H COSY (▬) and HMBC (→) correlations of Terrein (5)**.

A final interpretation of **5 **was carried out by HMBC experiment (Figure 3), fixing the structure as 4,5-dihydroxy-3-propenyl-cyclopent-2-enone; terrein [38,39]. Terrein (**5**) has a hypopigmentary effect in Mel-Ab cells, and it is a strongly down-regulator of melanin synthesis by reducing tyrosinase production [40], and inhibit human platelet aggregation [41]. Terrein showed a strong antiproliferative effect on skin equivalents [42] and as proteasome inhibitor and anti-tumoral drug [43].

#### 2.3.6. Butyrolactone-V (6)

The molecular weight of **6 **was established as 440 Dalton (C_24_H_24_O_8_), containing 13 double bond unsaturations. The ^1^H NMR/H,HCOSY spectrum of compound **6 **revealed the presence 1,4-disubstituted (*δ *7.54 and 6.85, *J *~ 8.8 Hz), and unsymmetrical tri-substituted (m, *δ *6.48) aromatic systems. A 1H dd signal (δ 5.02) of an oxygenated methine attached to a dd signal (δ 2.80) of a methylene group, confirming their ABX property. A singlet of methoxy group (δ 3.77), 2H methylene singlet (δ 3.40) flanked by two *sp*^2 ^systems, and two methyl singlets (δ 1.25 and 1.16), being of gem dimethyl groups were deduced.

Based on the ^13^C NMR/HMQC spectra of compound **6**, two carbonyls (*δ *171.5 and 170.3), two phenolic carbons (*δ *159.4 and 153.3), β-quaternary carbon (*δ *139.6) of an ester or lactone system were deduced. Two 2CH *sp*^2 ^signals (*δ *130.4, 116.6), and 3CH *sp*^2 ^signals (*δ *132.9, 130.4, 117.2), being of 1,4-disubstitued and unsymmetric tri-substituted phenolic residues were shown. Two *sp*^3 ^quaternary oxy-carbons (*δ *86.8 and 78.0), one oxy-methine (*δ *70.4), one carbomethoxy (*δ *53.9) and two methylenes (*δ *39.0, 2.80) were visible. Two gem dimethyl signals attached to an oxygenated quaternary carbon were displayed (*δ *25.9, 20.9).

Based on the above spectroscopic data and molecular formula, compound **6 **exhibited a strong close structural similarity to butyrolactone-I (**2**). In accordance, three structural formulas were proposed according to search in AntiBase: butyrolactone-V (**6**) [44,45], 4-hydroxy-2-[2-(1-hydroxy-1-methyl-ethyl)-2,3-dihydro-benzofuran-5-ylmethyl]-3-(4-hydroxy-phenyl)-5-oxo-2,5-dihydro-furan-2-carboxylic acid methyl ester (**8**) [21] and butyrolactone-III (**9**) [19].

**Figure 4:**
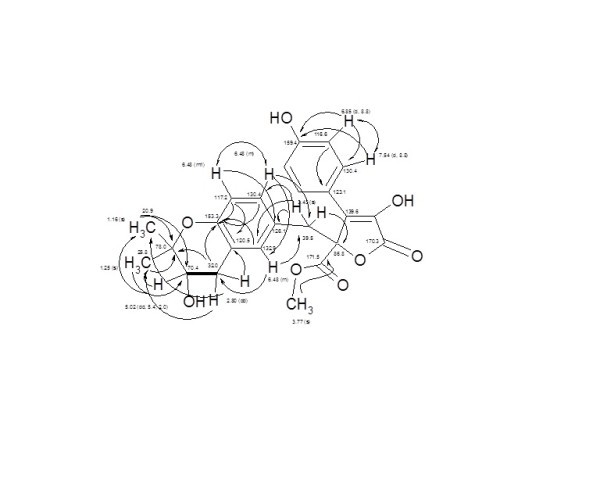
**Full H,H COSY (↔, ▬) and HMBC (→) correlations of Butyrolactone-V (6)**.

The structure was confirmed by detailed 2D experiments; H,H COSY and HMBC (Figure 4) and comparison with literature as butyrolactone-V (**6**) [25,42,43]. Butyrolactone-V was reported to exhibit a moderate antimalarial activity against the malarial parasite *Plasmodium falciparum *K1 (IC50 7.9 μg/mL) [45].

#### 2.3.7. 4,4'-Diydroxypulvinone (Aspulvinone E) (7)

Compound **7 **was obtained with a molecular weight of 296 Dalton (C_17_H_12_O_5_) by HRESI MS, bearing 12 double bond equivalents. The ^1^H NMR spectrum of **7 **showed five signals in the aromatic region for 9H (*δ *8.06-5.88), representing two 1,4-disubstitued phenolic residues (*J *~ 8.6 Hz). The fifth signal (1H) was shown as singlet at δ 5.88. Based on the revealed spectroscopic data and molecular formula and search in AntiBase, two alternatives, 4,4'-diydroxypulvinone (**7**) and Gyrocyanin (**10**) were displayed. However, the chemical shift of the singlet methine in compound **10 **has high field shifting (*δ *4.96), which was not matching with our revealed spectral data, establishing the structure as 4,4'-diydroxypulvinone (**7**) [46].

### 2.4. Biological activities

Activity patterns of the mycelial and supernatant extracts produced by fungal strain *A. flavipes *MM2 against set of microorganisms namely, *Staphylococcus aureus, Pseudomonas aeruginosa, Candida albicans *and *Aspergillus niger *were carried out using agar disc method (25 μg/disc, ∅ 5 mm). In accordance, both extracts showed high antibacterial (16-14 mm) and moderate anti-yeast and antifungal (10-14 mm) activities (Table 1).

**Table 1:** Pre-antimicrobial assays of *A. flavipes *MM2 (ϕ mm)

**Medium no**.	**Inhibition zone (mm)**							
	
	**Culture filtrate extract**				**Cells extract**			
	
	***St.*** ***aureus***	***Ps. aeruginosa,***	***C. albicans***	***A. niger***	***St. Aureus***	***Ps. aeruginosa,***	***C. albicans***	***A. niger***
	
Czapeck-Dox	15	16	14	12	14	14	10	10

Alternatively, the isolated compounds **1-7 **were tested against *Bacillus subtilis, S. aureus, Streptomyces viridochromogenes *(Tü 57), *Escherichia coli, C. albicans, Mucor miehi, Chlorella vulgaris, Chlorella sorokiniana, Scenedesmus subspicatus, Rhizoctonia solani *and *Pythium ultimum *(40 μ*g*/disc, ∅ 9 mm). According to this study, only four compounds (**1**, **2**, **4**, **7**) were active. Ergosterol (**1**) was highly and moderately active against *S. aureus *and *B. subtilis*. Compounds **7**, **4 **and **3 **showed high and moderate activity against *S. aureus*,. Finally, the whole studied compounds were tested against brine shrimp (10 μg/mL) for cytotoxic activities, exhibiting no cytotoxicity except ergosterol (**1**), which showed 100% cytotoxicity after 15 h (Table 2).

**Table 2:** Antimicrobial assays of *A. flavipes *MM2 compounds (ϕ mm)

Compound (No.)	Inhibition zone (mm) of tested microorganisms											
	
	*B.* *sub.*	*St.* *aur*	**St**. *Virid*	*E.* *coli*	*C.* *alb*	*M. miehi*	**Ch**. vulg	*Ch. Sorok*	*Sce.* *sub*	*R.* *solani*	*P.* *ultim*	Brine shrimp
Ergosterol (**1**)	11	19	ND	ND	ND	ND	ND	ND	ND	ND	ND	100%
Butyrolactone I (**2**)	ND	ND	ND	ND	ND	ND	ND	ND	ND	ND	ND	ND
Aspulvinone H (**3**)	ND	11	ND	ND	ND	ND	ND	ND	ND	ND	ND	ND
6-methylsalicylic acid (**4**)	ND	15	ND	ND	ND	ND	ND	ND	ND	ND	ND	ND
Terrien (**5**)	ND	ND	ND	ND	ND	ND	ND	ND	ND	ND	ND	ND
Butyrolactone-V (**6**)	ND	ND	ND	ND	ND	ND	ND	ND	ND	ND	ND	ND
4,4'-diydroxypulvinone (**7**)	ND	18	ND	ND	ND	ND	ND	ND	ND	ND	ND	ND

## 3. Experimental

NMR spectra were measured on Varian Unity 300 and Varian Inova 600 spectrometers. Electron spray ionization mass spectrometry (ESI HRMS): Finnigan LCQ ion trap mass spectrometer coupled with a Flux Instruments (Basel, Switzerland) quaternary pump Rheos 4000 and a HP 1100 HPLC (nucleosil column EC 125/2, 100-5, C 18) with autosampler (Jasco 851-AS, Jasco Inc., Easton, MD, USA) and a Diode Array Detector (Finnigan Surveyor LC System). High-resolution mass spectra (HRMS) were recorded by ESI MS on an Apex IV 7 Tesla Fourier-Transform Ion Cyclotron Resonance Mass Spectrometer (Bruker Daltonics, Billerica, MA, USA). EI MS at 70 eV with Varian MAT 731, Varian 311A, AMD-402, high-resolution with perflurokerosine as standard. *R*_f _values were measured on Polygram SIL F/UV_254 _(Merck, pre-coated sheets). Size exclusion chromatography was done on Sephadex LH-20 (Pharmacia).

### 3.1. Isolation and taxonomy of the producing strain

The terrestrial *A. flavipes *MM2, which was identified according to the Raper and Fennel [14], has been isolated from rice hulls sample by placing the rice hulls over water agar medium (g/L): Agar-agar (20) and water (100%) with incubation at 28°C for 7 days the developing colony was transferred to Czapeks agar with incubation at 28°C for 10 days. Bright whit-faint yellow colonies with a brown straining background of the fungal strain were grown. The colonies are growing rather slowly, showing whitish from conidial masses, with brownish conidiophores shining through, reverse yellow-brown to red brown Conidial heads spas, loosely columnar, conidiophores smooth-walled, pale yellow to light brown 2.4-3.2 μm diameter. According to its morphological and microscopic characteristics and comparison with literature, the fungal strain was assigned as *A. flavipes *MM2 [14]. The strain is deposited in Dr Mohammad Magdy El Metwally collection in Microbiology Department, Soil & Water and Environment Research Institute, ARC, Giza, Egypt.

### 3.2. Fermentation, extraction and isolation

Small pieces (1 cm^2^) of well grown sub-cultures of *A. flavipes *MM2 were inoculated into thirty 1-L Erlenmeyer flasks, each containing 300 mL of sterilized Czapeck-Dox medium (g/L): Sucrose (30), NaNO_3 _(3), K_2_HPO_4 _(1), KCl (0.5), MgSO_4 _(0.5), FeSO_4 _(0.01) and distilled water (1 L) at pH = (7.3). The inoculated flasks were incubated for 10 days at 28°C and 100 rpm. After harvesting, the fungal mate and supernatant were separated by filtration. The fungal mat was then applied to maceration in methanol (3 × 0.5 L). The methanol extract was concentrated in vacuum and the remaining aqueous solution was re-extracted by ethyl acetate followed by concentration to yield 845 mg as brown crude extract. The supernatant was passed through XAD-16 column (4 × 120 cm). After washing with water, the absorbed organic extract was eluted by methanol, followed by concentration under vacuum, and the aqueous residue was re-extracted by ethyl acetate, followed by concentration in vacuo to afford 818 mg as brown crude extract.

The mycelial extract (845 mg) was subjected to fractionation using silica gel column chromatography (cyclohexane-CH_2_Cl_2_-MeOH) to afford 200 mg as major fraction, which was then washed by MeOH to deliver a colourless precipitate. The last precipitate was purified on Sephadex LH-20 (CH_2_Cl_2_/40% MeOH) to afford Ergosterol (**1**, 12 mg) as colourless solid.

The filtrate crude extract (818 mg) was fractionated on silica gel column and eluted by cyclohexane-CH_2_Cl_2_-MeOH gradient to give five fractions (I-V). Fraction FIII was further fractionated using Sephadex LH-20 column (MeOH) to afford two sub-fractions, FIIIa (44 mg) and FIIIb (73 mg). Purification of FIIIa by Sephadex LH-20 (MeOH) afforded a colourless solid of butyrolactone-I (**2**, 4 mg). PTLC purification (CH_2_Cl_2_/5%MeOH) of FIIIb followed by Sephadex LH-20 (MeOH) afforded Aspulvinone H (**3**) as colourless solid (3 mg). A further fractionation of FIV on Sephadex LH-20 (MeOH) led to sub-fractions FIVa (35 mg) and FIVb (120 mg). Purification of sub-fraction FIVa using Sephadex LH-20 (MeOH), PTLC (CH_2_Cl_2_/5% MeOH), and then Sephadex LH-20 (MeOH) resulted in 6-methylsalicylic acid (**4**, 10 mg) as colourless solid. Sub-fraction FIVb was purified on Sephadex LH-20 (MeOH) to afford Terrien (**5**, 10 mg) and butyrolactone-V (**6**, 2 mg) as two colourless solids. Finally, fraction FV was purified using two subsequent Sephadex LH-20 columns (MeOH) to give a yellow solid of 4,4'-diydroxypulvinone (**7**, 4 mg).

#### 3.2.1. Ergosterol; ergosta-5,7,22-triene-3β-ol (1)

C_28_H_44_O (396), colourless solid, UV-absorbing, turned violet with anisaldehyde/sulphuric acid, *R*_f _= 0.46 (CH_2_Cl_2_/5%MeOH); **^1^H NMR **(CDCl_3_, 300 MHz): *δ *= 5.57 (dm, 1H, H-6), 5.38 (dm, 1H, H-7), 5.17 (m, 2H, H-22,23), 3.62 (m, 1H, H-3), 2.46 (dm, 1H, H-5), 2.35 (m, 2H, H-20, 24), 2.09-1.93 (m, 3H), 1.92-1.89 (m, 4H), 1.88-1.55 (m, 4H), 1.50-1.40 (m, 3H), 1.38-1.16 (m, 3H), 1.02 (d, *J *= 7.2, 3H, CH_3_-21), 0.93 (s, 3H, CH_3_-19), 0.91 (d, *J *= 7.2, 3H, CH_3_-28), 0.82 (d, *J *= 6.8, 3H, CH_3_-27), 0.80 (d, *J *= 6.8, 3H, CH_3_-26), 0.61 (s, 3H, CH_3_-18); **^13^C NMR **(CDCl_3_, 75 MHz): *δ *= 141.3 (C_q_-8), 139.8 (C_q_-5), 135.5 (CH-22), 131.9 (CH-23), 119.6 (CH-7), 116.3 (CH-6), 70.4 (CH-3), 55.7 (CH-17), 54.5 (CH-14), 46.2 (CH-9), 42.8 (C_q_-13), 42.8 (CH-24), 40.7 (CH_2_-4), 40.4 (CH-20), 39.1 (CH_2_-12), 38.4 (CH_2_-1), 37.0 (C_q_-10), 33.1 (CH-25), 32.0 (CH_2_-2), 28.3 (CH_2_-16), 23.0 (CH_2_-11), 21.1 (CH_2_-15), 21.1 (CH_3_-21), 19.9 (CH_3_-27), 19.6 (CH_3_-26), 17.6 (CH_3_-28), 16.2 (CH_3_-19), 12.0 (CH_3_-18); **EI-MS (70 EV): ***m/z *(%) = 396 ([M]^+^, 87), 378 ([M-H_2_O]^+^, 12), 363 ([M-(H_2_O+CH_3_)]^+^, 100), 271 (25), 253 (52), 211 (33).

#### 3.2.2. Butyrolactone-I (2)

C_24_H_24_O_7 _(424), colourless solid, UV-absorbing, turned violet with anisaldehyde/sulphuric acid, *R*_f _= 0.39 (CH_2_Cl_2_/5% MeOH); **^1^H NMR **(CD_3_OD, 300 MHz): *δ *= 7.57 (d, *J *= 8.8 Hz, 2H, H-2',6'), 6,86 (d, *J *= 8.8 Hz, 2H, H-3',5'), 6.50 (dd, *J *= 7.1, 2.0 Hz, 1H, H-6"), 6.48 (d, *J *= 8.1 Hz, 1H, H-5"), 6.40 (d, *J *= 1.7, 1H, H-2"), 5.05 (t, *J *= 3.7 Hz, 1H, H-8"), 3.76 (s, 3H, 7-OCH_3_), 3.42 (d, *J *= 3.9, 2H, CH_2_-5), 3.06 (d, *J *= 7.3 Hz, 2H, CH_2_-7"), 1.65 (s, 3H, CH_3_-10"), 1.56 (s, 3H, CH_3_-11"); **^13^C NMR **(CD_3_OD, 75 MHz): *δ *= 171.6 (C_q_-6), 170.3 (C_q_-1), 159.3 (C_q_-4'), 155.0 (C_q_-4"), 139.6 (C_q_-3), 133.0 (C_q_-9"), 132.4 (CH-2"), 130.4 (CH-2',6'), 129.8 (CH-6"), 129.2 (C_q_-3"), 125.0 (C_q_-1"), 123.1 (C_q_-1'), 123.6 (CH-8"), 116.6 (CH-3',5'), 115.0 (CH-5"), 86.8 (C_q_-4), 53.8 (OCH_3_-7), 39.6 (CH_2_-5), 28.7 (CH_2_-7"), 25.9 (CH_3_-10"), 17.8 (CH_3_-11"); **-(+)ESI MS**: *m/z *(%) = 447 ([M+Na]^+^, 81), 871 ([2M+Na]^+^, 100); **-(-)ESI MS**: *m/z *(%) = 423 ([M-H]^-^, 13), 847 ([2M-H]^-^, 4); **(+)-HRESI**: *m/z *= 447.1414 [M+Na]^+ ^(calc. 447.1414 for C_24_H_24_NaO_7_); **(-)-HRESI**: *m/z *423.1435 [M-H]^- ^(calc. 423.1449 for C_24_H_23_O_7_).

#### 3.2.3. Aspulvinone H (3)

C_27_H_28_O_5 _(432), colourless solid, UV-blue fluorescent, turned yellow with anisaldehyde/sulphuric acid, *R*_f _= 0.62 (CH_2_Cl_2_/10% MeOH), **^1^H NMR **(CD_3_OD, 300 MHz): *δ *= 7.81 (d, *J *= 1.8 Hz, 1H, H-2'), 7.68 (m, 1H, H-6'), 7.59 (m, 1H, H-6"), 7.44 (d, *J *= 1.6 Hz, H-2"), 6.73 (m, 2H, H-5',5"), 6.22 (s, H-5), 5.36 (m, 2H, H-8',8"), 3.40-3.00 (m, 4H, H_2a, b_-7', H_2a, b_-7"), 1.75 (m, 12H, H_3_-10',11',10",11"); **-(+)ESI MS**: *m/z *(%) = 455 ([M+Na]^+^, 56), 477 ([M+2Na-H]^+^, 100), 887 ([2M+Na]^+^, 5); **-(-)ESI MS**: *m/z *(%) = 431 ([M-H]^-^, 100), 863 ([2M-H]^-^, 4), **(+)-HRESI**: *m/z *455.1808 [M+Na]^+ ^(calc. 455.1829 for C_27_H_28_NaO_5_); **(-)-HRESI**: *m/z *431.1860 [M-H]^- ^(calc. 431.1864 for C_27_H_27_O_5_).

#### 3.2.4. 6-Methylsalicylic acid (4)

C_8_H_8_O_3 _(152), colourless solid, UV-absorbing, *R*_f _= 0.24 (CH_2_Cl_2_/5%MeOH); **^1^H NMR **(CD_3_OD, 300 MHz): *δ *= 7.07 (t, *J *= 7.7 Hz, 1H, 4-H), 6.63 (d, *J *= 8.2 Hz, 1H, 3-H), 6.59 (d, *J *= 7.5 Hz, 1H, 5-H), 2.56 (s, 3H, CH_3_-7); **^13^C NMR **(CD_3_OD, 75 MHz): *δ *= 176 (C_q_-8), 162.7 (C_q_-2), 142.7 (C_q_-6), 132.1 (CH-4), 122.9 (CH-3), 119.5 (C_q_-1), 115.1 (CH-5), 23.3 (CH_3_-7); **-(+)ESI MS**: *m/z *(%) = 175 ([M+Na]^+^, 25), 371 ([2M+3Na-2H]^+^, 55); **-(-)ESI MS**: *m/z *(%) = 151 ([M-H]^-^, 100), 303 ([2M-H]^-^, 4).

#### 3.2.5. Terrine (5)

C_8_H_10_O_3 _(154), colourless solid, UV-absorbing, turned dark green on spraying with anisaldehyde/sulphuric acid, *R*_f _= 0.51 (CH_2_Cl_2_/10%MeOH); **^1^H NMR **(CD_3_OD, 300 MHz): *δ *= 6.82 (ddd, *J *= 13.7, 8.9, 6.8 Hz, 1H, H-2), 6.42 (dd, *J *= 15.8, 1.1 Hz, 1H, H-3), 5.99 (s, 1H, H-5), 4.67 (d, *J *= 2.4 Hz, 1H, H-8), 4.07 (d, *J *= 2.7 Hz, 1H, H-7), 1.97 (dd, *J *= 6.8, 1.4 Hz, 3H, 1-CH_3_); **^13^C NMR **(CD_3_OD, 75 MHz): *δ *= 205.6 (C_q_-6), 170.8 (C_q_-4), 141.8 (CH-2), 126.4 (CH-3), 125.9 (CH-5), 82.4 (CH-7), 78.1 (CH-8), 19.5 (CH_3_-1); **-(+)ESI MS**: *m/z *(%) = 177 ([M+Na]^+^, 62), 331 ([2M+Na]^+^, 100); **-(-)ESI MS**: *m/z *(%) = 153 ([M-H]^-^, 34), 307 ([2M-H]^-^, 4); **(+)-HRESI MS**: *m/z *177.0528 [M+Na]^+ ^(calc. 177.0522 for C_8_H_10_NaO_3_); **(-)-HRESI MS**: *m/z *153.0553 [M-H]^- ^(calc. 153.0557 for C_8_H_9_O_3_).

#### 3.2.6. Butyrolactone-V (6)

C_24_H_24_O_8 _(440), colourless solid, UV-absorbing, turned pink with anisaldehyde/sulphuric acid, *R*_f _= 0.12 (CH_2_Cl_2_/5% MeOH), **^1^H NMR **(CD_3_OD, 300 MHz): *δ *= 7.54 (d, *J *= 8.8 Hz, 2H, H-2',6'), 6.85 (d, *J *= 8.8 Hz, 2H, H-3',5'), 6.48 (m, 3H, H-2",5",6"), 5.02 (dd, *J *= 5.2, 2.0 Hz, 1H, H-8"), 3.77 (s, 3H, OCH_3_-7), 3.40 (s, 2H, CH_2_-5), 2.80 (dd, *J *= 5.2, 16.9 Hz, 2H, CH_2_-7"), 1.25 (s, 3H, CH_3_-10"), 1.16 (s, 3H, CH_3_-11"); **^13^C NMR **(CD_3_OD, 75 MHz): *δ *= 171.5 (C_q_-6), 170.3 (C_q_-1), 159.4 (C_q_-4'), 153.3 (C_q_-4"), 139.6 (C_q_-3), 132.9 (CH-2"), 130.4 (CH-2',6'), 130.4 (CH-6"), 120.5 (C_q_-3"), 126.0 (C_q_-1"), 123.1 (C_q_-1'), 116.6 (CH-3',5'), 117.2 (CH-5"), 86.8 (C_q_-4), 78.0 (C_q_-9"), 70.4 (CH-8"), 53.9 (OCH_3_-7), 39.5 (CH_2_-5), 32.0 (CH_2_-7"), 25.8 (CH_3_-10"), 20.9 (CH_3_-11"); **-(+)ESI MS**: *m/z *(%) = 441 ([M+H]^+^, 30), 463 ([M+Na]^+^, 57.5), 881 ([2M+H]^+^, 25), 903 ([2M+Na]^+^, 50); **-(-)ESI MS**: *m/z *(%) 439 ([M-H]^-^, 3); **(+)-HRESI**: *m/z *463.1370 [M+Na]^+ ^(calc. 463.1363 for C_24_H_24_NaO_8_); **(-)-HRESI**: *m/z *439.1399 [M-H]^- ^(calc. 439.1389 for C_24_H_23_O_8_).

#### 3.2.7. 4,4'-Diydroxypulvinone (7)

C_17_H_12_O_5 _(296), colourless solid, UV yellow fluorescence, *R*_f _= 0.15 (CH_2_Cl_2_/5% MeOH); **^1^H NMR **(DMSO-d_6_, 300 MHz): *δ *= 9.42 (brs, 1H, 4'-OH), 8.70 (brs, 1H, 4"-OH), 8.06 (d, *J *= 8.6 Hz, 2H, H-2',6'), 7.51 (d, *J *= 8.7 Hz, 2H, H-2",6"), 6.75 (d, *J *= 8.7 Hz, 2H, H-3', 5'), 6.16 (d, *J *= 8.7 Hz, 2H, H-3", 5"), 5.88 (s, 1H, H-5); **^13^C NMR **(DMSO-d_6_, 300 MHz): *δ *= 156.1 (C_q_-4',4"), 152.4 (C_q_-4), 130.4 (CH-2',2",6',6"), 126.1 (C_q_-1', 1"), 124.6 (CH-5), 115.3 (CH-3',5'), 114.1 (CH-3",5"); **-(+)-ESI MS**: *m/z *(%) = 319 ([M+Na]^+^, 20), 314 ([M+2Na-H]^+^, 30); **-(-)ESI MS**: *m/z *(%) = 295 ([M-H]^-^, 100); (+)-**HRESI MS: ***m/z *319.0587 [M+Na]^+ ^(calc. 319.0577 for C_17_H_12_NaO_5_). **(-)-HRESI**: *m/z *295.0616 [M-H]^- ^(calc. 295.0612 for C_17_H_11_O_5_).

### 3.3. Biological activities

#### 3.3.1. Antimicrobial activity

Antimicrobial assays were conducted utilizing the disc-agar method [47]. This has been carried out gainst diverse sets of microorganisms. *A. flavipes *MM2 extract was dissolved in CH_2_Cl_2_/10% MeOH at a concentration of 1 mg/mL. Aliquots of 40 μL were soaked on filter paper discs (9 mm ∅, no. 2668, Schleicher & Schüll, Germany) and dried for 1 h at room temperature under sterilized conditions. The paper discs were placed on inoculated agar plats and incubated for 24 h at 38°C for bacterial and 48 h (30°C) for the fungal isolates, while the algal test strains were incubated at approximately 22°C in day light for 8-10 days. The pure compounds were examined against the test microorganisms: *B. subtilis, S. aureus, S. viridochromogenes *(Tü 57), *E. coli, C. albicans, M. miehi, C. vulgaris, C. sorokiniana, S. subspicatus, R. solani *and *P. ultimum*.

#### 3.3.2. Brine shrimp microwell cytotoxicity assay

The cytotoxic assay was performed according to Sajid et al.'s screening [48].

## Competing interests

The authors declare that they have no competing interests.
